# New mtDNA Association Model, MutPred Variant Load, Suggests Individuals With Multiple Mildly Deleterious mtDNA Variants Are More Likely to Suffer From Atherosclerosis

**DOI:** 10.3389/fgene.2018.00702

**Published:** 2019-01-08

**Authors:** Agnieszka Piotrowska-Nowak, Joanna L. Elson, Agnieszka Sobczyk-Kopciol, Aleksandra Piwonska, Aleksandra Puch-Walczak, Wojciech Drygas, Rafal Ploski, Ewa Bartnik, Katarzyna Tonska

**Affiliations:** ^1^Faculty of Biology, Institute of Genetics and Biotechnology, University of Warsaw, Warsaw, Poland; ^2^Institute of Genetic Medicine, Newcastle University, Newcastle upon Tyne, United Kingdom; ^3^Centre for Human Metabolomics, North-West University, Potchefstroom, South Africa; ^4^Department of General Biology and Parasitology, Medical University of Warsaw, Warsaw, Poland; ^5^Department of Epidemiology, Cardiovascular Disease Prevention and Health Promotion, Institute of Cardiology, Warsaw, Poland; ^6^Department of Prevention and Education, Department of Arterial Hypertension and Diabetology, Medical University of Gdansk, Gdansk, Poland; ^7^Department of Medical Genetics, Medical University of Warsaw, Warsaw, Poland; ^8^Institute of Biochemistry and Biophysics Polish Academy of Sciences, Warsaw, Poland

**Keywords:** mtDNA variation, MutPred, variant load, common complex diseases, atherosclerosis

## Abstract

The etiology of common complex diseases is multifactorial, involving both genetic, and environmental factors. A role for mitochondrial dysfunction and mitochondrial DNA (mtDNA) variation has been suggested in the pathogenesis of common complex traits. The aim of this study was to investigate a potential role of mtDNA variants in the development of obesity, diabetes, and atherosclerosis in the Polish population. Whole mtDNA sequences from 415 Polish individuals representing three disease cohorts and a control group were obtained using high-throughput sequencing. Two approaches for the assessment of mtDNA variation were applied, traditional mitochondrial haplogroup association analysis and the mutational or variant load model using the MutPred pathogenicity prediction algorithm for amino acid substitutions in humans. We present a possible association between mildly deleterious mtDNA variant load and atherosclerosis that might be due to having more than one likely mildly deleterious non-synonymous substitution. Moreover, it seems largely dependent upon a few common haplogroup associated variants with MutPred score above 0.5.

## Introduction

The growing worldwide prevalence of obesity, diabetes and atherosclerosis, common complex traits associated with modern lifestyle, has a high socioeconomic cost, making solving their pathomechanisms a pressing problem. The etiology of common complex diseases is by definition multifactorial, involving both genetic and environmental factors, as well as the interaction between the two. A role for mitochondrial dysfunction in many common complex, metabolic, and degenerative diseases has been suggested and investigated in many studies (Wallace, 2005, 2013). Supporting this, mitochondria are central to pathways crucial for cellular homeostasis (Wallace, 2013; Venter et al., 2018).

Mitochondrial DNA (mtDNA) is maternally inherited and variation acquired throughout the evolution of mtDNA has defined the emergence of distinct lineages in the human population, called haplogroups (Chinnery and Schon, 2003; Elson and Lightowlers, 2006). Although mtDNA is small, it encodes 13 respiratory chain proteins and 24 RNAs necessary for their translation, all indispensable for energy production through the oxidative phosphorylation (Anderson et al., 1981). Mutations of mtDNA are an important cause of inherited disease (Gorman et al., 2016). An epidemiological study in the North East of England established minimum prevalence of adult mtDNA disease as 1 in 5,000 demonstrating that as a group they are among the most common inherited human diseases (Gorman et al., 2015). MtDNA mutations cause a variety of disorders that affect tissues with high energy demands, such as Leber hereditary optic neuropathy (LHON) and mitochondrial encephalopathy, lactic acidosis, and stroke-like episodes (MELAS) (Gorman et al., 2016). Mitochondrial diseases are clinically heterogeneous and can result in a broad range of symptoms, including diabetes mellitus and cardiomyopathy (Wallace, 2013). Interestingly, the m.3243A>G mutation in one of the mitochondrial tRNA Leu genes, which is associated primarily with MELAS (~80% of cases), can also cause another syndrome called maternally inherited diabetes and deafness (MIDD) (Gorman et al., 2016). However, the m.3243A>G mutation manifesting as MIDD explains < 0.5% of type 2 diabetes cases (in UK Caucasians) (Saker et al., 1997).

Many studies have suggested that the role of mtDNA variation in human health is not limited to inherited mitochondrial disorders, but that mtDNA population variation might contribute to the pathogenesis of common complex diseases (Herrnstadt and Howell, 2004; Wallace, 2010). The mitochondrial genome was implied to contribute to their missing heritability (Hudson et al., 2014). Pedigree analysis in families affected with diabetes showed that inheritance in the maternal line was more frequently observed than in the paternal line (Lyssenko et al., 2015), which suggests a contribution of the mtDNA in diabetes pathogenesis. Obesity is a well-known phenotype associated with the lifestyle of food overconsumption and physical inactivity that might be ameliorated or exacerbated by inherited genetic variants that play a role in metabolic pathways. It has been speculated that biochemical differences in mitochondrial respiratory chain function may predispose to obesity. These differences have been postulated to result from different efficiency of coupling of electron transport to ATP synthesis by the respiratory chain, that is the efficiency of converting protons from nutrients to ATP. Tightly coupled mitochondria produce maximum amount of ATP per calorie consumed with minimum energy dispersed as heat (Wallace, 2005). Uncoupling of the mitochondrial oxidative phosphorylation system was observed to prevent obesity in mice under a high-calorie diet (Li et al., 2000). This suggests that stronger mitochondrial coupling might be a risk factor for obesity as fewer calories and thus less food would be required to produce the same amount of ATP, therefore leading to fat storage in case of excessive food intake. Obesity is also one of the best recognized risk factors for type 2 diabetes mellitus (T2DM). Both conditions can lead to serious cardiovascular complications, such as atherosclerosis and myocardial infarction. One of the notable pathomechanisms of atherosclerosis links this cardiovascular disease with mitochondrial function. It implies the essential role of oxidative stress and inflammatory state due to increased levels of reactive oxygen species (ROS) and the mitochondrial respiratory chain is considered the major site of ROS production. Progress of inflammation in the walls of large arteries contributes to formation and deposition of lipid plaques and change of arterial structure eventually leading to closing of the lumen (Matsuda and Shimomura, 2013; Venter et al., 2018). Inflammation has also been postulated in obesity where elevated levels of systemic oxidative stress have been reported, and diabetes where oxidative stress induced by hyperglycemia has been implicated in vascular complications and pancreatic β-cell failure (Matsuda and Shimomura, 2013).

The mitochondrial genome has a high level of sequence variation with different populations being characterized by (region) specific patterns of polymorphisms, which define mitochondrial haplogroups (Torroni et al., 1996; Wallace et al., 1999; Herrnstadt et al., 2002). Mitochondrial haplogroup association study is one of the most common approaches to investigate the role of mtDNA variation in human complex diseases. This hypothesis is similar to the “common-disease common-variant” hypothesis where common genetic variants found deep within the phylogeny are investigated. Significant association of a disease with one mtDNA haplogroup suggests that a common variant or variants of mtDNA associated with the haplogroup contribute to disease development as risk or protective factors.

Besides looking for common mtDNA variants affecting the risk of a common complex disease or course of that disease, a hypothesis considering the cumulative effect of multiple mostly rare mildly deleterious variants (a “rare-variant common-disease” hypothesis) is gaining interest (Elson et al., 2006; Pienaar et al., 2017; Venter et al., 2017). A rare variant may be a risk factor in isolation or combined with more rare variants or a common polymorphism. Since natural selection removes variants that are mildly deleterious from the population, they are likely to be rare and more frequent in younger branches of mtDNA phylogeny (Elson et al., 2004; Pereira et al., 2011; Soares et al., 2013; Wei et al., 2017). Thanks to the next generation sequencing methods that allow high throughput sequencing in a cost-effective and time-saving manner studies that sequence the complete mitochondrial chromosome can, and should, now be done routinely to investigate the role of mtDNA variation in complex traits.

One approach to using complete mtDNA sequence data is to make an estimate of the cumulative impact of a person's non-synonymous mtDNA variation, which can be done using a variety of methodologies. One such method is to use the MutPred algorithm for the prediction of the impact of amino acid substitutions in humans. This tool has been well-validated in the mitochondrial context having been utilized in the assessment of mtDNA variation in a number of studies (Pereira et al., 2011, 2012; Pienaar et al., 2017; Venter et al., 2017). MtDNA association studies based on complete mtDNA sequence data, and investigating the role of high (>0.5) MutPred scoring variants is considered a good approach as these variants are predicted to affect protein function (Pienaar et al., 2017; Venter et al., 2017).

In view of the information outlined above, we addressed the issue of genetic predisposition to the common complex diseases by investigating the role of mtDNA variation in obesity, T2DM and atherosclerosis with the traditional mitochondrial haplogroup association approach and with the mutational or variant load model using the MutPred tool. The presented work investigated the contribution of inherited mtDNA population variants, somatic variation was not the object of this study.

## Materials and Methods

### Sample Collection

DNA samples used in this study were collected from peripheral blood of 415 Polish individuals, aged 27–82 years, representing three disease cohorts and a control group. The three patient populations consisted of 100 participants each and included individuals with a) diagnosed T2DM (according to criteria meeting Polish Diabetes Association recommendations) and preferably with possible maternal inheritance; b) obesity based on BMI index above 30 (and preferably >35) without diabetes; and c) atherosclerosis enrolled by previous myocardial infarction (preferably at a young age and below 50 y/o for men and 60 y/o for women) which is most commonly caused by atherosclerosis. The 115 control subjects were matched for ethnicity, gender, and age to patients and recruited based on the following inclusion criteria: (a) glycaemia within normal range (3.9–5.5 mmol/l) and no diabetes; (b) BMI within normal range (18.5–24.99); and (c) no history of myocardial infarction in subjects and their parents. Case-control studies are not always capable of capturing the continuous process of disease development, apparent in many complex traits and particularly in atherosclerosis. Thus, in this study one of the final clinical consequences of the disease, myocardial infarction, served as an indicator of the degree of disease progression instead of a disease inclusion/exclusion criterion. Samples included in this study were collected in two research centers: Department of Medical Genetics, Medical University of Warsaw (T2DM) and Department of Epidemiology, Cardiovascular Disease Prevention and Health Promotion, Institute of Cardiology (obesity, atherosclerosis and controls) with the latter subjects selected from the Multi-Center National Population Health Examination Survey (WOBASZ study) (Drygas et al., 2016).

### Ethical Statements

All procedures on human genetic material and data within this study were performed in accordance with the ethical principles of the local Ethics Committee of the Medical University of Warsaw (approval number KB/264/2012) and the Declaration of Helsinki. Informed consent was obtained from all individual participants prior to the inclusion in the study.

### mtDNA Sequencing and Analysis

Whole mitochondrial genome amplification for sequencing was performed on total DNA of each subject as one or two overlapping fragments using the long-range PCR. Sequences and positions of both primer sets are depicted in Supplemental Table 1. PCR reactions were performed according to the rapid protocol of PrimeSTAR GXL DNA Polymerase kit (TaKaRa Bio Inc., Kusatsu, Japan) with minor modifications: primers at a final concentration of 0.2 μM each, 0.3125 U of polymerase, up to 100 ng of genomic DNA as a template and a 25-μl volume reaction. 3-step PCR with 3 and 1.5-min extension time was used for single and two amplicons, respectively. The annealing temperature was set to 64°C for single PCR product, 59 and 62°C for products A and B, respectively. PCR products were purified using 0.6 × volume of Agencourt AMPure XP magnetic beads (Beckman Coulter, Brea, USA) and quantified with Qubit dsDNA High Sensitivity Assay Kit and Qubit fluorometer (Thermo Fisher Scientific, Waltham, USA). Indexed mtDNA libraries were processed for sequencing using Nextera XT DNA Sample Preparation Kit and Nextera XT Index Kit (Illumina Inc., San Diego, USA) according to the manufacturer's protocol. Massively parallel sequencing was performed on the Illumina MiSeq instrument with a 301-cycle paired-end read chemistry, MiSeq Reagent Kit v3 (Illumina Inc., San Diego, USA).

Base calling, demultiplexing and FASTQ file generation were automatically performed on the MiSeq platform (Illumina Inc., San Diego, USA). Quality control of sequencing reads, alignment to the human mtDNA reference sequence (rCRS, NC_012920) and variant detection were performed using CLC Genomics Workbench software (CLC bio, Aarhus, Denmark). The mitochondrial haplogroup was predicted for each participant using Mitomaster, an online mtDNA sequence analysis tool (Lott et al., 2013), based on a full set of SNVs. A more detailed description of the methodology used here can be found in a paper by Piotrowska-Nowak et al. (2019). The variant population frequency was obtained using a dataset of over 30 000 human mtDNA sequences deposited in GenBank and available from the MITOMAP database (Lott et al., 2013). Variants with a GenBank frequency < 0.1% were assigned as rare.

MutPred provides a prediction of the probability of deleterious amino acid substitution based on its impact on the evolutionary conservation, structural and functional properties of protein (Li et al., 2009; Pereira et al., 2011; Hepp et al., 2015). It has numerical scoring system with 0–1 value range. Variants with a score above 0.5 are considered as potentially deleterious to protein function (“actionable hypothesis” that the molecular mechanism would be disrupted) and those with a score above 0.75 as high confidence harmful changes (Li et al., 2009; Pereira et al., 2011). MutPred scores were assigned to all operationally homoplasmic non-synonymous substitutions of each subject using mtDNA Server (Weissensteiner et al., 2016). The effect of heteroplasmic mtDNA variants was not investigated here. MutPred mutational load of each patient's sequence—here referred to as “variant load” to clarify that what is being measured is the burden of predicted mildly deleterious variants in individuals in case and control cohorts—was calculated as the sum of MutPred scores for all non-synonymous variants detected in a sample, as described previously (Pienaar et al., 2017; Venter et al., 2017). In this study only variants with high MutPred scores, that is above the 0.5 threshold, were used for calculations as they are more likely to be deleterious and thus are suggested to give a clearer indication of a pathogenic load (Pienaar et al., 2017; Venter et al., 2017).

The 415 mitochondrial sequences investigated in this study are available at GenBank under accession numbers MH120425-MH120839.

### Statistical Analysis

The differences in the mitochondrial haplogroup distribution between disease cohorts and the control group were investigated by Fisher's exact test. *Post-hoc* analysis of the contingency table using the adjusted standardized residual (adj. std. res.) method was carried out to determine the relationship. Strength of the association for statistically significant outcome was estimated using Phi coefficient (ϕ).

A one-way ANOVA followed by a Dunnett's *post-hoc* test was used to compare mean MutPred derived mildly deleterious variant loads in diabetic, obese and atherosclerotic patients to the control group. The frequency of rare mtDNA variants (with GenBank frequency below 0.1%) was examined between each of the three disease groups and controls separately using Fisher's exact test. The prevalence of participants having variants with MutPred score above 0.5 or 0.7 was compared among disease cohorts and control subjects with Fisher's exact test. The number of individuals having one or more high scoring variants (that is with MutPred score above 0.5) in each phenotype cohort was compared to the control group using Fisher's exact test. A Bonferroni correction of *p*-value for multiple testing was applied where appropriate. The statistical analysis was performed using IBM SPSS Statistics software (IBM Corporation, Armonk, USA). The results were considered statistically significant when the *p*-value was < 0.05.

## Results

In the current study, mtDNA variation in common complex diseases was analyzed using the case-control approach. Characteristics of the studied cohorts are shown in Table 1. All obese participants had BMI score above 33.38 of T2DM and 36 of atherosclerosis patients also had BMI above 30. Previous myocardial infarction was also noted for 18 diabetes subjects, whereas 2 atherosclerosis patients were also diagnosed with diabetes.

**Table 1:** Characteristics of the complex trait patients and the control group.

**Parameter**	**T2DM**	**Obesity**	**Atherosclerosis**	**Controls**
Number of participants (n)	100	100	100	115
Women	50	50	50	59
Men	50	50	50	56
Mean age ± SD (year)	59.3 ± 10.10	54.7 ± 9.26	55.5 ± 7.30	56.7 ± 9.49
BMI ± SD	29.7 ± 6.58^a^	38.5 ± 5.22	29.1 ± 5.20	22.7 ± 1.81
Fasting glucose levels ± SD (mmol/l)	n/a	5.8 ± 2.01^b^	5.4 ± 0.88^c^	4.8 ± 0.40

a *For 3 T2DM patients BMI data were unavailable*.

b *For 2 obese patients fasting glucose data were unavailable*.

c *For 1 atherosclerosis patient fasting glucose data were unavailable*.

### Proven Pathogenic Mutations

We examined disease patients and control individuals for the presence of proven pathogenic variants in mtDNA. None of the subjects had the m.3243A>G mutation which has been associated with the MIDD syndrome. We found in total 2 individuals (1 obese and 1 with atherosclerosis) with the m.1555A>G mutation at a heteroplasmic (11.81%) and homoplasmic level, respectively, that is associated with aminoglycoside-induced and non-syndromic sensorineural deafness transmitted in a maternal manner.

### Mitochondrial Haplogroup Distribution

In total, 15 different mitochondrial haplogroups were identified in the studied cohorts, their observed frequencies are summarized in Table 2. No statistically significant differences were noted in T2DM (Fisher's exact test value 13.213, *p* = 0.319), obesity (16.736, *p* = 0.154) or atherosclerosis (18.203, *p* = 0.128) when compared for total haplogroup composition with the common control group. However, *post-hoc* analysis of the contingency table using the adjusted standardized residual method indicated lower than expected prevalence of haplogroup V in T2DM patients when compared to the control group (1 vs. 7%, adj. std. res. −2.18, *p* = 0.02961, ϕ = −0.148). Moreover, the test residuals revealed also that fewer obese and atherosclerotic subjects were assigned to haplogroup U when compared to controls (12 vs. 26 %, adj. std. res. −2.60, *p* = 0.00936, ϕ = −0.177 for obesity; 15 vs. 26 %, adj. std. res. −1.99, *p* = 0.04624, ϕ = −0.136 for atherosclerosis). None of the *post-hoc* analysis *p*-values was, however, significant after Bonferroni correction for multiple testing.

**Table 2:** Prevalence of mitochondrial haplogroups in the studied cohorts.

**Mitochondrial DNA haplogroup**	**T2DM patients (*n* = 100)**	**Obesity patients (*n* = 100)**	**Atherosclerosis patients (*n* = 100)**	**Control subjects (*n* = 115)**
C	1	0	1	1
D	0	1	0	2
G	0	0	1	0
H	42	37	40	43
HV	0	1	1	2
I	5	2	3	1
J	9	13	10	7
K	4	6	3	6
N	0	2	2	0
R	1	0	0	2
T	10	14	14	8
U	22	12	15	30
V	1	6	3	8
W	3	3	6	3
X	2	3	1	2

### Rare mtDNA Variants

We identified in total 498, 506, 531, and 543 non-synonymous mtDNA variants in patients with T2DM, obesity, atherosclerosis and control individuals, respectively. We specified variants present exclusively in each of the studied groups (unique variants) in order to have a clear distinction between groups and increase possible signal. Variant population frequency was determined based on the prevalence in mtDNA sequences in the GenBank database. To test if frequency of rare non-synonymous variants differs between each of the disease cohorts and control subjects, and in particular if rare variants are overrepresented in any of the studied phenotypes, we compared the numbers of rare (< 0.1%) and common (>1%) non-synonymous variants. We did not find any differences between any of the disease cohorts and controls, as shown in Table 3.

**Table 3:** Number of rare (<0.1%) and common (>1%) non-synonymous variants unique to T2DM, obesity, atherosclerosis, and the control group.

**GenBank frequency**	**Disease group**	**Control group**	***P*-value**
**T2DM**			
<0.1%	22	27	1
>1%	3	5
**OBESITY**			
<0.1%	14	28	1
>1%	4	9
**ATHEROSCLEROSIS**			
<0.1%	26	28	0.675
>1%	2	4

*P-value from Fisher's exact test*.

### MutPred Variant Load

**Figure 1:**
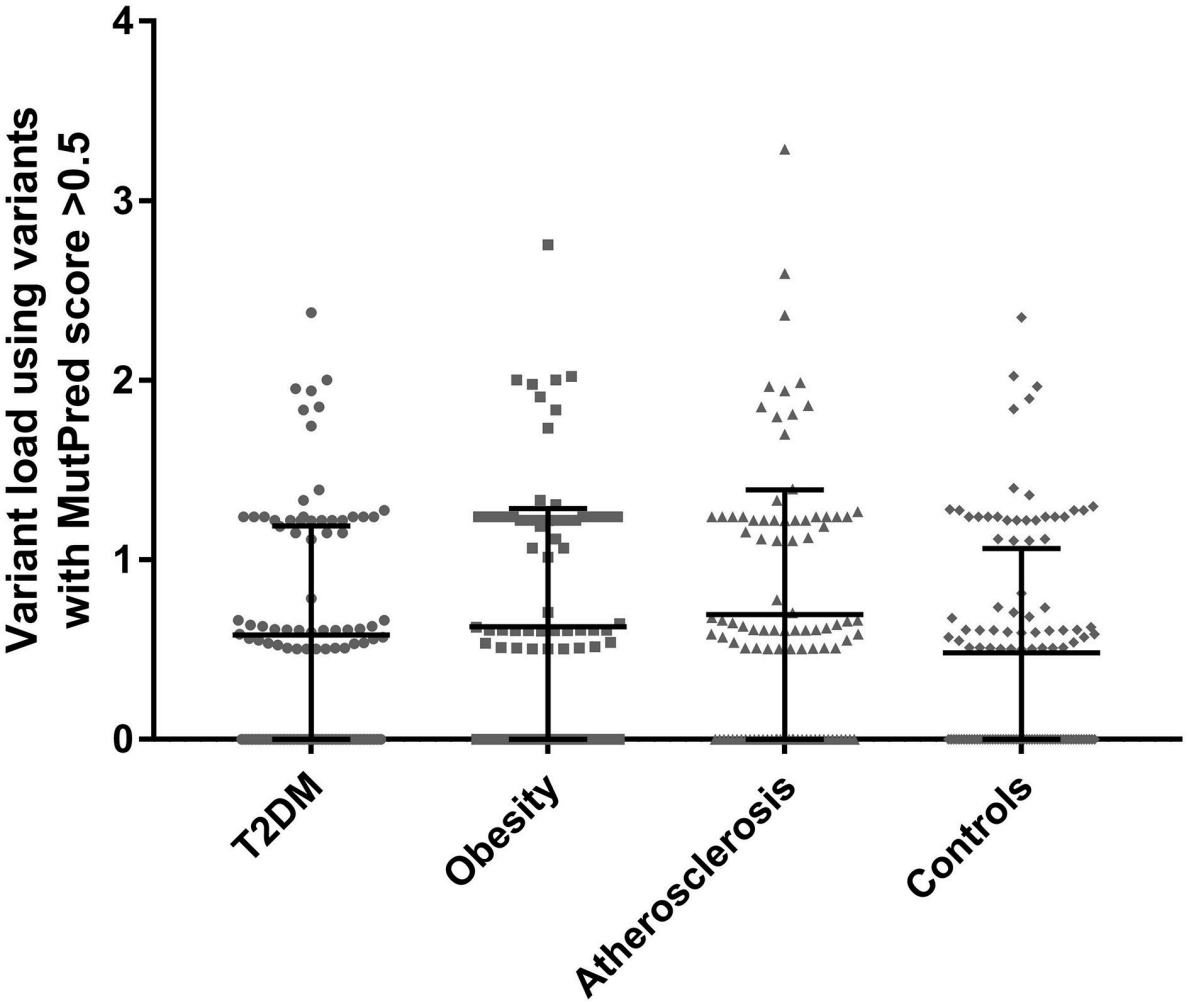
Total variant loads based on only non-synonymous variants with MutPred score above 0.5 threshold in T2DM (*n* = 100), obesity (*n* = 100), atherosclerosis (*n* = 100) and the control group (*n* = 115). No significant differences were observed when comparing all groups with one-way ANOVA (*p* = 0.096). Dunnett's *post-hoc* test revealed a significant difference between the atherosclerosis cohort and the control group (*p* = 0.041) in mean total variant load.

Total variant loads using variants with MutPred scores above >0.5 were calculated for each participant in the studied cohorts and are presented in Figure 1. For the exact variant load values with the number of non-synonymous substitutions see the Supplemental Tables 2–5. To test whether higher MutPred derived mildly deleterious variant load might be an indication of a complex trait we compared total loads between all groups using one-way ANOVA. Although all three disease groups had higher mean variant load levels than controls (means with SD for T2DM, obesity, atherosclerosis and control cohorts were 0.58 ± 0.61, 0.63 ± 0.66, 0.70 ± 0.70, and 0.48 ± 0.58, respectively), no significant differences were observed (*p* = 0.096). However, Dunnett's two-tailed *post-hoc* test used to compare each disease phenotype to the single common control group indicated significantly higher variant load in atherosclerosis patients (*p* = 0.041). None of the remaining groups was found to be different from controls.

Next, we tested if disease cohorts differ in the number of variants with high (>0.5) and very high (>0.7) MutPred scores when compared to controls. The number of subjects within the studied groups carrying mtDNA variants above 0.5 or 0.7 MutPred threshold is listed in Table 4. We observed that atherosclerosis patients were more likely to have high pathogenicity scored variants when compared to controls (65% of atherosclerosis group and 50% of controls, *p* = 0.038). However, the difference was not robust enough to withstand correction for multiple comparisons and, moreover, the significance was lost when the 0.7 MutPred threshold was applied (Table 4). This can be attributed to the small number of individuals having variants with MutPred scores >0.7. None of the remaining groups was found to be different from controls. We also investigated if having more than one variant with high MutPred score (>0.5) might be associated with disease development. The number of individuals in each disease phenotype was compared to controls by frequency of variants that is having 0, 1, or 2+ non-synonymous sequence changes with above 0.5 pathogenic score. As shown in Table 5, none of the disease groups were observed to differ significantly from controls in variant number distribution.

**Table 4:** Frequency of complex trait and control subjects having high scoring (>0.5 or >0.7) mtDNA variants with MutPred analysis.

**MutPred threshold**	**Disease group**	**Control group**	***P*-value**
**T2DM**			
>0.5	59	58	0.220
≤0.5	41	57
>0.7	8	11	0.811
≤0.7	92	104
**OBESITY**			
>0.5	58	58	0.276
≤0.5	42	57
>0.7	8	11	0.811
≤0.7	92	104
**ATHEROSCLEROSIS**			
>0.5	65	58	0.038
≤0.5	35	57
>0.7	10	11	1
≤0.7	90	104

*P-value from Fisher's exact test*.

**Table 5:** Number of subjects having one or more high scoring (>0.5) mtDNA variants with MutPred analysis.

**Number of variants with MutPred score >0.5**	**Number of disease subjects**	**Number of control individuals**	***P*-value**
**T2DM**			
0	41	57	0.434
1	30	31
2+	29	27
**OBESITY**			
0	42	57	0.134
1	22	31
2+	36	27
**ATHEROSCLEROSIS**			
0	35	57	0.083
1	31	31
2+	34	27

*P-value from Fisher's exact test*.

**Figure 2:**
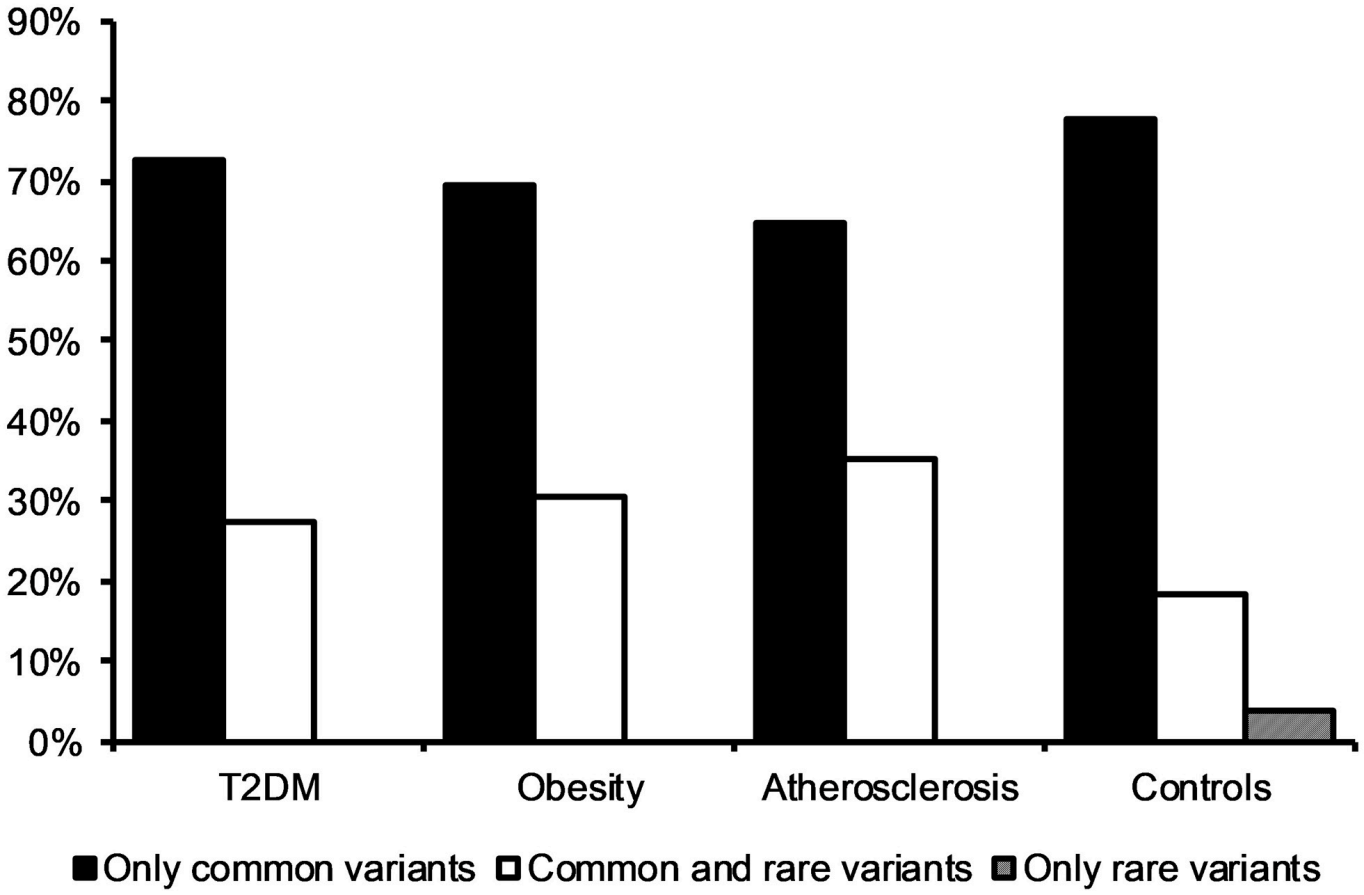
Frequency of subjects having two or more mtDNA variants with MutPred score above 0.5 in disease cohorts and the control group. The majority of individuals had a combination of two or more common variants only.

Observing that there is a tendency for having multiple rather than single mtDNA variants with MutPred score above 0.5 in disease cohorts when compared to the control group, we investigated the variants in more detail. Interestingly, we noted that several common population variants associated with different mtDNA haplogroups were still present in our subjects even with the high MutPred score threshold (Table 6). Common population mtDNA variants that are associated with haplogroups, besides rare variants, have been observed to have high MutPred scores also by other authors, e.g., Venter et al. (2017). However, significance of such variation has been debated and whether they exert a phenotypic effect may be dependent on genetic background, e.g. haplogroup context (Queen et al., 2017; O'Keefe et al., 2018). Additionally, the majority of subjects having 2 or more variants with MutPred score above 0.5, had a combination of relatively common variants only, as presented in Figure 2. Those with atherosclerosis were noted to have the highest number of subjects with a combination of a common and a rare (not frequently associated with a particular haplogroup) variants in comparison to other cohorts (Figure 2).

**Table 6:** Common variants with MutPred score above 0.5 and associated with mtDNA haplogroups found in disease cohorts and the control group.

**Mitochondrial variant**	**MutPred score**	**GenBank frequency**	**Number of T2DM sequences**	**Number of obesity sequences**	**Number of atherosclerosis sequences**	**Number of control sequences**	**Associated haplogroups^a^**	**Comment**
m.3394T>C	0.783	1.36%	1	4	0	0	8	haplogroup associated
m.4216T>C	0.611	10.01%	20	27	24	18	14	haplogroup defining^b^
m.4917A>G	0.628	4.75%	10	14	14	8	5	haplogroup defining^b^
m.5460G>A	0.505	6.23%	6	7	10	8	36	haplogroup associated
m.8414C>T	0.513	4.01%	0	1	0	2	1	haplogroup associated
m.8584G>A	0.553	5.01%	1	0	1	1	5	haplogroup associated
m.11969G>A	0.639	1.34%	0	0	1	1	5	haplogroup associated
m.12811T>C	0.587	1.08%	1	0	1	0	3	haplogroup associated
m.13780A>G	0.606	1.76%	5	4	4	1	1	haplogroup associated
m.14798T>C	0.609	6.76%	12	17	10	11	5	haplogroup associated
m.15204T>C	0.547	1.25%	0	0	0	1	4	haplogroup associated
m.15257G>A	0.785	1.61%	1	1	2	2	3	haplogroup associated
m.15812G>A	0.518	1.02%	0	0	1	0	3	haplogroup associated

a *Number of hits in Phylotree*.

b *Based on haplogroup markers from Mitomap database*.

## Discussion

Mitochondrial dysfunction and inherited mtDNA variation have been implicated in the development of common complex cardiovascular and metabolic diseases (Wallace, 2013; Venter et al., 2018). In this study we investigated mtDNA variation in Polish subjects with obesity, diabetes and atherosclerosis to test whether it might have an impact on disease susceptibility in this population. Screening for the presence of proven pathogenic variants in mtDNA revealed only one change, m.1555A>G, which is associated with deafness, present in two subjects. The m.1555A>G mutation was reported at the frequency of 0.4% in the general Polish population (Rydzanicz et al., 2009). Considering the similar prevalence of m.1555A>G variant in our study (0.48%, 2/415) and the known reduced penetrance of hearing loss in individuals who are not exposed to aminoglycoside antibiotics (Usami and Nishio, 1993), we think this finding is incidental.

Many studies have reported mitochondrial haplogroup associations with complex traits, but a number of problems with this model have been shown (Salas and Elson, 2015). A single haplogroup-associated common variant might have occurred more than once in different branches of the mtDNA phylogeny, which complicates haplogroup association studies (Herrnstadt et al., 2002). Moreover, such analysis investigates common polymorphic variation rather than rare variants, which are most likely to be mildly deleterious. Complications in haplogroup studies are also caused by a high level of population stratification when considering mtDNA variation, due to mtDNA's small effective population size (Salas and Elson, 2015; Pienaar et al., 2017). As a result, many studies fail to replicate findings from similar prior studies (Chinnery et al., 2007; Knoll et al., 2014), resulting in a myriad of conflicts in the literature. Having this in mind, we investigated the distribution of mitochondrial haplogroups in our cohorts and found that mitochondrial haplogroup V was underrepresented in T2DM, whereas haplogroup U was underrepresented in obesity and atherosclerosis. The significances of these associations were, however, not robust enough to withstand correction for multiple comparisons, thus a protective role of two mtDNA haplogroups in the development of the studied complex traits could not be confirmed.

We then tested if rare (GenBank frequency below 0.1%) non-synonymous substitutions were more common in cases than controls, we found no significant differences across studied groups. However, this negative result is based solely on population frequency data, and not all variants will be mildly deleterious, some will one day become common. Thus, we adopted a mutational or variant load model and used the MutPred tool to predict the impact of non-synonymous substitutions, to ensure the best possible predictive model was applied.

The MutPred variant load method presented here focuses on population variants with a score above 0.5 that are predicted to be mildly deleterious to protein function, and thus tend to be rare variants being found at the tips of the phylogeny. These rare variants are less likely to be impacted on by population stratification (Elson et al., 2004; Pereira et al., 2011; Venter et al., 2017), with population stratification thought to be a cause of false associations in prior haplogroup based association studies. By using only mtDNA variants with a MutPred score above 0.5, the effect of the reference sequence on the phylogeny is also largely mitigated, as reported in the paper of Venter et al. (2017), which showed the number of variants in sequences of African and European background were similar under this condition. Additionally, low scoring variants are more common, and of questionable impact thus excluding them from the analysis aims also at reducing likely background noise in the analysis. Using this method, we noted a borderline significant difference in atherosclerosis patients that had higher MutPred derived mildly deleterious variant loads. Moreover, they were more likely to have at least one high (>0.5) pathogenicity scored non-synonymous substitution than controls (Table 4), yet the statistical significance of this finding was not robust enough to withstand correction for multiple testing. Overall, we have not observed affected individuals to be more likely to have multiple high scoring non-synonymous variants, however, there was a marginally significant result in the atherosclerosis group (Table 5). Combined with the ANOVA analysis, our results suggest that if mtDNA variation is involved in atherosclerosis development, individuals are likely to require more than one mildly deleterious variant to be more susceptible to the disease. Investigating larger cohorts would be better powered to detect such synergistic effect as individuals with two or more such variants only represent around 30% of the population.

It is important to mention that not all of the variants with MutPred scores >0.5 are rare, we observed some common population mtDNA polymorphisms to have a high MutPred score (above 0.5, Table 6). Moreover, we noted that very few subjects have two or more rare high scoring variants. Thus, we hypothesize that rare mtDNA variants acting in synergy within individuals having any of the studied phenotypes is an unlikely to be a common element in their etiology. Rather, the presence of multiple high scoring common haplogroup associated variants was the most prevalent configuration where two variants of predicted mildly deleterious effect were seen. In the context of interpreting these results it is worth considering the evidence that mitochondrial haplogroup context may influence the impact or penetrance of clinically proven mtDNA mutations (Chinnery et al., 1999; Hudson et al., 2007; Queen et al., 2017; Wei et al., 2017), as such it might be possible that the impact of more common variants is also dependent on haplogroup background making these observations complex to interpret. That is a high scoring common variant might have been miss-assigned the high value by the MutPred algorithm, or it might be appropriately scored but the deleterious effect might be masked by other variants on the lineage where it is commonly found.

Interestingly, atherosclerosis subjects had the highest tendency to have at least one common haplogroup associated variant and a rare variant with a MutPred score >0.5 when compared to other groups. This lends some supports to a two-hit hypothesis (Knudson, 1971) that is when acquiring another mildly deleterious variant if already having a high scoring common haplogroup variant might result in some subtle change to risk of atherosclerosis. One of the strengths of the MutPred load measure being that it is able to detect the synergistic effect between high-scoring common variants and rare variants, and such an interaction might have resulted in the borderline effect observed in this study.

In summary, we present an advance on the mutational or variant load hypothesis for the study of the role of mtDNA variation in common complex traits. Although the MutPred load method is designed to be less vulnerable to the effects of population stratification, we showed in our study that this measure is not completely independent of haplogroup context as it will lead to the inclusion of some common haplogroup associated variants. Moreover, we present a possible association between mildly deleterious mtDNA variants and atherosclerosis that might be due to a subset of individuals having more than one likely mildly deleterious variant and that seems largely dependent upon a few common haplogroup variants with MutPred score above 0.5. Particular emphasis is laid on the possibility of a synergistic effect or interplay of common mtDNA haplogroup associated variants with MutPred scores >0.5 in combination with rarer variants of similar MutPred score in disease susceptibility. These mildly deleterious non-synonymous mtDNA variants, especially acting in synergy, might influence the function of the respiratory chain. A possible mild change in the function of subunits of its complexes may in consequence lead to the increase in ROS production that would, together with other risk factors, contribute to the disease development. For full discussion on the putative role of mitochondria and mtDNA in the etiology of cardiovascular diseases we recommend a paper by Venter et al. (2018) and Yu et al. (2012).

## Author Contributions

AP-N carried out the experimental work, provided sequence data, conducted analysis, and wrote the manuscript. JE thought of the work concept and design, managed project and collaboration, and revised manuscript providing critical comments. AS-K selected patient samples and revised the manuscript. AP, AP-W and WD managed WOBASZ project and revised the manuscript. RP managed collaboration and revised the manuscript. EB managed project and revised manuscript providing critical comments. KT managed project, collaboration and funding, and revised manuscript providing critical comments. All authors have approved the final article.

### Conflict of Interest Statement

The authors declare that the research was conducted in the absence of any commercial or financial relationships that could be construed as a potential conflict of interest.
