# Supplementary material for: New mtDNA Association Model, MutPred Variant Load, Suggests Individuals With Multiple Mildly Deleterious mtDNA Variants Are More Likely to Suffer From Atherosclerosis
Source: Front Genet. 2019 Jan 8;9:702. doi: 10.3389/fgene.2018.00702 (PMC6332467; doi:10.3389/fgene.2018.00702)
Supplement: Supplementary file 1 [file Data_Sheet_1.docx]

**SUPPLEMENTAL REFERENCES**

Zhang, W., Cui, H., and Wong, L.-J. C. (2012). Comprehensive one-step molecular analyses of mitochondrial genome by massively parallel sequencing. *Clin. Chem.* 58, 1322–1331. doi:10.1373/clinchem.2011.181438.

**SUPPLEMENTAL TABLES**

| **Supplemental Table 1.** Primers for mtDNA amplification | | | | |
| --- | --- | --- | --- | --- |
| Primer | Fragment | Sequence | Position in mtDNA^b^ | Product length (bp) |
| mt16426F | single amplicon^a^ | 5’-CCGCACAAGAGTGCTACTCTCCTC-3’ | 16426-16449 | 16,569 |
| mt16425R |  | 5’-GATATTGATTTCACGGAGGATGGTG-3’ | 16425-16401 |  |
| mt132F | A | 5’-CTTTGATTCCTGCCTCATCC-3’ | 132-151 | 8,531 |
| mt8662R |  | 5’-GGGTGGTGATTAGTCGGTTG-3’ | 8662-8643 |  |
| mt8467F | B | 5’CCTACCTCCCTCACCAAAGC-3’ | 8467-8486 | 8,927 |
| mt824R |  | 5’-ATCACTGCTGTTTCCCGTGG-3’ | 824-805 |  |

^a^(Zhang et al., 2012)

^b^According to the revised Cambridge Reference Sequence (rCRS) of mtDNA (GenBank: NC_012920)

*F* forward, *R* reverse

| Supplemental Tables 2-5. Variant loads using only variants with MutPred score >0.5 for all participants in the study | | |
| --- | --- | --- |
| T2DM subject index | Total variant load using variants with MutPred score >0.5 | Number of variants with MutPred score >0.5 |
| D105 | 1.220 | 2 |
| D109 | 0.562 | 1 |
| D116 | 0.609 | 1 |
| D132 | 0.664 | 1 |
| D136 | 1.239 | 2 |
| D137 | 0.000 | 0 |
| D142 | 0.000 | 0 |
| D145 | 0.000 | 0 |
| D147 | 2.003 | 3 |
| D195 | 0.000 | 0 |
| D199 | 0.559 | 1 |
| D2 | 0.000 | 0 |
| D200 | 0.000 | 0 |
| D205 | 0.000 | 0 |
| D21 | 0.630 | 1 |
| D218 | 0.000 | 0 |
| D22 | 0.000 | 0 |
| D223 | 0.609 | 1 |
| D233 | 1.746 | 3 |
| D237 | 0.616 | 1 |
| D240 | 0.000 | 0 |
| D249 | 0.000 | 0 |
| D250 | 1.390 | 2 |
| D252 | 1.239 | 2 |
| D266 | 0.000 | 0 |
| D268 | 1.151 | 2 |
| D276 | 0.000 | 0 |
| D277 | 0.000 | 0 |
| D282 | 0.000 | 0 |
| D285 | 0.539 | 1 |
| D317 | 0.000 | 0 |
| D32 | 0.609 | 1 |
| D326 | 0.537 | 1 |
| D327 | 0.000 | 0 |
| D328 | 0.000 | 0 |
| D329 | 0.785 | 1 |
| D332 | 0.000 | 0 |
| D337 | 1.239 | 2 |
| D339 | 0.000 | 0 |
| D34 | 1.239 | 2 |
| D343 | 0.596 | 1 |
| D344 | 0.000 | 0 |
| D348 | 0.000 | 0 |
| D350 | 0.505 | 1 |
| D352 | 0.000 | 0 |
| D353 | 0.000 | 0 |
| D354 | 0.527 | 1 |
| D366 | 1.331 | 2 |
| D371 | 0.000 | 0 |
| D372 | 0.614 | 1 |
| D376 | 1.220 | 2 |
| D378 | 1.220 | 2 |
| D380 | 1.115 | 2 |
| D382 | 0.000 | 0 |
| D383 | 1.239 | 2 |
| D392 | 0.000 | 0 |
| D402 | 0.505 | 1 |
| D405 | 1.836 | 3 |
| D414 | 1.943 | 3 |
| D418 | 0.509 | 1 |
| D422 | 1.219 | 2 |
| D424 | 0.000 | 0 |
| D43 | 0.553 | 1 |
| D432 | 0.000 | 0 |
| D435 | 0.569 | 1 |
| D437 | 0.533 | 1 |
| D44 | 0.000 | 0 |
| D444 | 1.239 | 2 |
| D454 | 0.000 | 0 |
| D46 | 1.220 | 2 |
| D460 | 0.000 | 0 |
| D468 | 1.220 | 2 |
| D469 | 0.000 | 0 |
| D479 | 0.505 | 1 |
| D49 | 0.000 | 0 |
| D491 | 1.151 | 2 |
| D509 | 0.630 | 1 |
| D514 | 1.188 | 2 |
| D526 | 1.277 | 2 |
| D53 | 0.000 | 0 |
| D534 | 1.220 | 2 |
| D54 | 0.505 | 1 |
| D544 | 0.000 | 0 |
| D547 | 0.000 | 0 |
| D552 | 0.587 | 1 |
| D58 | 0.000 | 0 |
| D63 | 1.239 | 2 |
| D65 | 1.853 | 3 |
| D67 | 2.377 | 4 |
| D68 | 0.611 | 1 |
| D74 | 0.000 | 0 |
| D77 | 0.000 | 0 |
| D78 | 0.509 | 1 |
| D83 | 0.509 | 1 |
| D87 | 0.000 | 0 |
| D91 | 0.638 | 1 |
| D94 | 0.664 | 1 |
| O64_21 | 1.151 | 2 |
| O64_85 | 0.611 | 1 |
| O70_87 | 1.955 | 3 |
| Obesity subject index | Total variant load using variants with MutPred scores >0.5 | Number of variants with MutPred scores >0.5 |
| O1_68 | 0.000 | 0 |
| O12_47 | 2.003 | 3 |
| O14_43 | 0.608 | 1 |
| O17_30 | 0.505 | 1 |
| O17_39 | 1.187 | 2 |
| O19_10 | 0.708 | 1 |
| O19_65 | 0.000 | 0 |
| O2_31 | 0.000 | 0 |
| O2_82 | 0.509 | 1 |
| O21_7 | 0.000 | 0 |
| O23_84 | 0.000 | 0 |
| O23_86 | 0.000 | 0 |
| O23_99 | 1.239 | 2 |
| O24_56 | 1.066 | 2 |
| O25_100 | 1.978 | 3 |
| O25_17 | 0.000 | 0 |
| O25_32 | 0.000 | 0 |
| O26_26 | 1.239 | 2 |
| O26_86 | 0.000 | 0 |
| O28_40 | 0.000 | 0 |
| O29_23 | 0.000 | 0 |
| O29_98 | 1.239 | 2 |
| O3_59 | 0.000 | 0 |
| O3_7 | 0.505 | 1 |
| O3_94 | 0.000 | 0 |
| O30_40 | 0.000 | 0 |
| O31_88 | 1.239 | 2 |
| O32_16 | 0.000 | 0 |
| O33_20 | 0.000 | 0 |
| O33_55 | 0.509 | 1 |
| O35_31 | 0.535 | 1 |
| O35_40 | 0.000 | 0 |
| O36_71 | 0.000 | 0 |
| O37_11 | 1.239 | 2 |
| O37_44 | 0.000 | 0 |
| O37_69 | 1.239 | 2 |
| O37_73 | 0.609 | 1 |
| O38_34 | 0.000 | 0 |
| O39_27 | 0.000 | 0 |
| O39_7 | 1.239 | 2 |
| O4_4 | 0.000 | 0 |
| O4_63 | 0.000 | 0 |
| O4_83 | 1.908 | 3 |
| O40_78 | 2.754 | 4 |
| O40_8 | 0.609 | 1 |
| O41_27 | 1.239 | 2 |
| O42_62 | 0.000 | 0 |
| O42_82 | 1.066 | 2 |
| O43_9 | 0.000 | 0 |
| O44_24 | 0.606 | 1 |
| O44_37 | 0.000 | 0 |
| O44_4 | 0.606 | 1 |
| O44_45 | 1.733 | 3 |
| O44_53 | 0.606 | 1 |
| O44_99 | 0.000 | 0 |
| O45_32 | 0.609 | 1 |
| O45_8 | 1.220 | 2 |
| O45_92 | 0.609 | 1 |
| O45_93 | 1.220 | 2 |
| O48_14 | 0.646 | 1 |
| O48_41 | 1.836 | 3 |
| O48_44 | 0.604 | 1 |
| O48_8 | 0.000 | 0 |
| O49_79 | 1.220 | 2 |
| O49_90 | 0.000 | 0 |
| O5_86 | 0.000 | 0 |
| O50_69 | 1.239 | 2 |
| O53_63 | 2.022 | 3 |
| O54_35 | 1.116 | 2 |
| O54_48 | 1.220 | 2 |
| O54_50 | 0.000 | 0 |
| O55_12 | 1.308 | 2 |
| O55_63 | 0.000 | 0 |
| O55_70 | 1.220 | 2 |
| O55_75 | 1.219 | 2 |
| O55_88 | 1.239 | 2 |
| O56_41 | 0.606 | 1 |
| O56_7 | 0.000 | 0 |
| O56_82 | 0.626 | 1 |
| O57_43 | 0.540 | 1 |
| O57_45 | 0.505 | 1 |
| O57_71 | 0.000 | 0 |
| O58_44 | 0.000 | 0 |
| O58_75 | 1.331 | 2 |
| O58_98 | 0.000 | 0 |
| O59_10 | 1.220 | 2 |
| O59_20 | 1.220 | 2 |
| O59_87 | 0.517 | 1 |
| O60_18 | 0.000 | 0 |
| O61_11 | 0.000 | 0 |
| O61_39 | 0.000 | 0 |
| O61_41 | 2.003 | 3 |
| O61_64 | 0.000 | 0 |
| O61_7 | 0.513 | 1 |
| O64_1 | 1.221 | 2 |
| O64_87 | 1.239 | 2 |
| O69_26 | 0.000 | 0 |
| O69_58 | 1.239 | 2 |
| O7_11 | 0.000 | 0 |
| O8_86 | 1.015 | 2 |
| Atherosclerosis subject index | Total variant load using variants with MutPred scores >0.5 | Number of variants with MutPred scores >0.5 |
| A1_43 | 1.239 | 2 |
| A1_80 | 0.630 | 1 |
| A10_59 | 0.505 | 1 |
| A12_57 | 0.000 | 0 |
| A12_63 | 0.639 | 1 |
| A12_71 | 0.000 | 0 |
| A12_76 | 0.000 | 0 |
| A14_74 | 0.505 | 1 |
| A14_92 | 1.396 | 2 |
| A18_67 | 1.239 | 2 |
| A18_86 | 0.705 | 1 |
| A2_20 | 2.363 | 4 |
| A2_8 | 1.219 | 2 |
| A20_31 | 0.649 | 1 |
| A24_26 | 0.606 | 1 |
| A24_67 | 0.000 | 0 |
| A24_73 | 1.239 | 2 |
| A25_38 | 0.509 | 1 |
| A26_41 | 0.000 | 0 |
| A26_47 | 0.779 | 1 |
| A27_58 | 0.000 | 0 |
| A27_68 | 0.609 | 1 |
| A27_70 | 1.220 | 2 |
| A28_47 | 1.701 | 3 |
| A28_48 | 2.596 | 4 |
| A28_6 | 0.540 | 1 |
| A32_61 | 1.239 | 2 |
| A32_67 | 0.588 | 1 |
| A32_91 | 0.000 | 0 |
| A33_32 | 0.000 | 0 |
| A33_35 | 1.239 | 2 |
| A33_59 | 1.811 | 3 |
| A33_87 | 1.239 | 2 |
| A34_16 | 0.000 | 0 |
| A34_67 | 0.000 | 0 |
| A34_79 | 1.124 | 2 |
| A35_74 | 0.000 | 0 |
| A35_83 | 0.505 | 1 |
| A36_4 | 0.609 | 1 |
| A36_91 | 0.000 | 0 |
| A38_100 | 1.220 | 2 |
| A38_99 | 0.000 | 0 |
| A39_26 | 0.610 | 1 |
| A39_64 | 1.188 | 2 |
| A4_31 | 1.860 | 3 |
| A4_77 | 0.610 | 1 |
| A40_63 | 1.106 | 2 |
| A40_95 | 0.000 | 0 |
| A42_64 | 1.220 | 2 |
| A44_85 | 1.270 | 2 |
| A49_55 | 0.000 | 0 |
| A51_63 | 0.506 | 1 |
| A52_5 | 1.220 | 2 |
| A52_65 | 0.000 | 0 |
| A52_67 | 0.000 | 0 |
| A54_12 | 1.331 | 2 |
| A55_61 | 0.000 | 0 |
| A57_100 | 0.000 | 0 |
| A58_18 | 0.000 | 0 |
| A58_4 | 1.941 | 3 |
| A59_45 | 0.000 | 0 |
| A59_56 | 1.156 | 2 |
| A60_43 | 1.966 | 3 |
| A60_7 | 0.000 | 0 |
| A64_39 | 1.852 | 3 |
| A64_53 | 0.000 | 0 |
| A65_13 | 0.569 | 1 |
| A66_3 | 0.000 | 0 |
| A66_68 | 0.000 | 0 |
| A67_60 | 0.505 | 1 |
| A67_95 | 0.509 | 1 |
| A68_62 | 0.609 | 1 |
| A70_54 | 0.620 | 1 |
| A70_66 | 1.797 | 3 |
| A70_94 | 0.000 | 0 |
| A71_38 | 1.116 | 2 |
| A71_6 | 0.609 | 1 |
| A72_42 | 1.239 | 2 |
| A72_55 | 0.000 | 0 |
| A72_9 | 0.553 | 1 |
| A73_38 | 0.000 | 0 |
| AO12_72 | 3.288 | 5 |
| AO26_87 | 0.000 | 0 |
| AO30_6 | 1.220 | 2 |
| AO35_47 | 0.000 | 0 |
| AO41_10 | 0.660 | 1 |
| AO43_49 | 0.000 | 0 |
| AO43_77 | 0.664 | 1 |
| AO44_23 | 0.664 | 1 |
| AO48_73 | 0.509 | 1 |
| AO56_45 | 1.239 | 2 |
| AO57_52 | 0.000 | 0 |
| AO58_39 | 0.000 | 0 |
| AO62_36 | 0.509 | 1 |
| AO62_94 | 0.000 | 0 |
| AO63_4 | 1.987 | 3 |
| AO65_72 | 0.679 | 1 |
| AO66_62 | 1.106 | 2 |
| AO71_69 | 0.000 | 0 |
| O47_56 | 0.587 | 1 |
| Control subject index | Total variant load using variants with MutPred scores >0.5 | Number of variants with MutPred scores >0.5 |
| K1_56 | 0.000 | 0 |
| K12_12 | 0.000 | 0 |
| K13_38 | 0.551 | 1 |
| K15_5 | 0.000 | 0 |
| K18_72 | 0.000 | 0 |
| K20_43 | 0.594 | 1 |
| K23_19 | 0.000 | 0 |
| K23_41 | 0.000 | 0 |
| K23_81 | 0.736 | 1 |
| K23_92 | 0.000 | 0 |
| K27_28 | 0.000 | 0 |
| K27_35 | 0.505 | 1 |
| K27_67 | 2.350 | 4 |
| K27_69 | 1.966 | 3 |
| K29_40 | 0.000 | 0 |
| K30_95 | 0.000 | 0 |
| K33_45 | 0.000 | 0 |
| K34_27 | 0.000 | 0 |
| K34_39 | 0.000 | 0 |
| K34_48 | 1.239 | 2 |
| K34_77 | 0.625 | 1 |
| K35_35 | 0.588 | 1 |
| K35_6 | 0.000 | 0 |
| K38_5 | 0.000 | 0 |
| K40_48 | 1.239 | 2 |
| K42_28 | 0.609 | 1 |
| K43_81 | 1.239 | 2 |
| K43_97 | 0.509 | 1 |
| K44_39 | 0.000 | 0 |
| K45_22 | 1.220 | 2 |
| K47_91 | 0.000 | 0 |
| K47_94 | 0.684 | 1 |
| K48_39 | 0.606 | 1 |
| K48_6 | 0.000 | 0 |
| K5_76 | 0.509 | 1 |
| K50_71 | 0.000 | 0 |
| K51_7 | 0.000 | 0 |
| K52_50 | 0.000 | 0 |
| K53_86 | 0.000 | 0 |
| K53_88 | 0.814 | 1 |
| K54_33 | 1.106 | 2 |
| K54_72 | 0.610 | 1 |
| K55_68 | 1.116 | 2 |
| K56_18 | 0.000 | 0 |
| K57_42 | 0.000 | 0 |
| K58_36 | 0.000 | 0 |
| K58_49 | 0.000 | 0 |
| K59_57 | 1.277 | 2 |
| K59_61 | 1.239 | 2 |
| K59_95 | 1.282 | 2 |
| K6_39 | 0.000 | 0 |
| K60_53 | 0.000 | 0 |
| K61_10 | 0.000 | 0 |
| K61_16 | 0.609 | 1 |
| K61_2 | 0.596 | 1 |
| K61_25 | 0.000 | 0 |
| K61_29 | 1.277 | 2 |
| K61_43 | 0.000 | 0 |
| K61_53 | 1.239 | 2 |
| K61_67 | 1.362 | 2 |
| K61_90 | 0.000 | 0 |
| K61_94 | 0.000 | 0 |
| K61_97 | 0.676 | 1 |
| K62_15 | 1.220 | 2 |
| K62_57 | 0.609 | 1 |
| K62_58 | 1.401 | 2 |
| K62_64 | 0.735 | 1 |
| K62_68 | 0.501 | 1 |
| K62_79 | 1.277 | 2 |
| K62_8 | 0.000 | 0 |
| K62_87 | 0.000 | 0 |
| K62_88 | 1.899 | 3 |
| K62_96 | 0.000 | 0 |
| K63_24 | 0.000 | 0 |
| K63_45 | 0.505 | 1 |
| K63_6 | 0.512 | 1 |
| K63_79 | 2.024 | 3 |
| K64_14 | 0.000 | 0 |
| K64_16 | 1.840 | 3 |
| K64_37 | 0.000 | 0 |
| K64_44 | 0.513 | 1 |
| K64_68 | 0.708 | 1 |
| K64_86 | 0.000 | 0 |
| K65_66 | 0.000 | 0 |
| K65_76 | 0.600 | 1 |
| K65_77 | 0.610 | 1 |
| K65_96 | 0.000 | 0 |
| K66_2 | 0.571 | 1 |
| K66_57 | 0.505 | 1 |
| K67_40 | 0.569 | 1 |
| K67_6 | 0.000 | 0 |
| K68_16 | 0.000 | 0 |
| K68_25 | 0.000 | 0 |
| K68_59 | 1.116 | 2 |
| K68_85 | 0.000 | 0 |
| K69_59 | 1.106 | 2 |
| K7_78 | 0.000 | 0 |
| K7_79 | 0.000 | 0 |
| K70_10 | 0.000 | 0 |
| K70_67 | 0.000 | 0 |
| K71_10 | 0.000 | 0 |
| K71_2 | 1.220 | 2 |
| K71_23 | 1.240 | 2 |
| K71_36 | 0.000 | 0 |
| K71_7 | 0.000 | 0 |
| K71_92 | 0.000 | 0 |
| K72_18 | 1.299 | 2 |
| K72_37 | 1.239 | 2 |
| K72_46 | 1.220 | 2 |
| K72_73 | 0.000 | 0 |
| K72_98 | 0.540 | 1 |
| K73_14 | 0.000 | 0 |
| K73_21 | 0.512 | 1 |
| K8_78 | 0.509 | 1 |
| K8_93 | 0.000 | 0 |
